# Farmworker Exposure to Pesticides: Methodologic Issues for the Collection of Comparable Data

**DOI:** 10.1289/ehp.8531

**Published:** 2006-02-16

**Authors:** Thomas A. Arcury, Sara A. Quandt, Dana B. Barr, Jane A. Hoppin, Linda McCauley, Joseph G. Grzywacz, Mark G. Robson

**Affiliations:** 1 Department of Family and Community Medicine, Wake Forest University School of Medicine, Winston-Salem, North Carolina, USA; 2 Division of Public Health Sciences, Wake Forest University School of Medicine, Winston-Salem, North Carolina, USA; 3 National Center for Environmental Health, Centers for Disease Control and Prevention, Atlanta, Georgia, USA; 4 Epidemiology Branch, National Institute of Environmental Health Sciences, National Institutes of Health, Department of Health and Human Services, Research Triangle Park, North Carolina, USA; 5 School of Nursing, University of Pennsylvania, Philadelphia, Pennsylvania, USA; 6 School of Public Health, University of Medicine and Dentistry of New Jersey, Piscataway, New Jersey, USA

**Keywords:** biomonitoring, data collection, environmental assessment, farmworker, health outcomes, pesticide exposure

## Abstract

The exposure of migrant and seasonal farmworkers and their families to agricultural and residential pesticides is a continuing public health concern. Pesticide exposure research has been spurred on by the development of sensitive and reliable laboratory techniques that allow the detection of minute amounts of pesticides or pesticide metabolites. The power of research on farmworker pesticide exposure has been limited because of variability in the collection of exposure data, the predictors of exposure considered, the laboratory procedures used in analyzing the exposure, and the measurement of exposure. The Farmworker Pesticide Exposure Comparable Data Conference assembled 25 scientists from diverse disciplinary and organizational backgrounds to develop methodologic consensus in four areas of farmworker pesticide exposure research: environmental exposure assessment, biomarkers, personal and occupational predictors of exposure, and health outcomes of exposure. In this introduction to this mini-monograph, first, we present the rationale for the conference and its organization. Second, we discuss some of the important challenges in conducting farmworker pesticide research, including the definition and size of the farmworker population, problems in communication and access, and the organization of agricultural work. Third, we summarize major findings from each of the conference’s four foci—environmental exposure assessment, biomonitoring, predictors of exposure, and health outcomes of exposure—as well as important laboratory and statistical analysis issues that cross-cut the four foci.

The exposure of migrant and seasonal farmworkers to pesticides at work and at home is an issue of continuing concern for public health (Arcury and Quandt 2003; Villarejo 2003). Accurately documenting exposure levels in this population is the focus of research programs across the nation. However, research on farmworker pesticide exposure is hampered by methodologic limitations and a lack of consensus on measures that should be used. On 30 September and 1 October 2004, a group of 25 scientists met to document the methodologic problems faced in farmworker pesticide exposure research and develop consensus on how these methodologic problems can be addressed.

The Farmworker Pesticide Exposure Comparable Data Conference was convened at the Graylyn International Conference Center, Wake Forest University, Winston-Salem, North Carolina. The conference participants included scientists of diverse disciplinary backgrounds employed by universities, private research organizations, governmental agencies, and industry. Participants included anthropologists, chemists, epidemiologists, nurses, physicians, psychologists, statisticians, and toxicologists. The articles in this mini-monograph document the results of this conference.

## Conference Rationale

Domestic and international research on pesticide exposure has increased dramatically since 1990. This research documents extensive occupational pesticide exposure among agricultural workers and their families (Acquavella et al. 2004; Curl et al. 2002; Eskenazi et al. 2004; Fenske et al. 2002; Lambert et al. 2005; Lu et al. 2000; Mandel et al. 2005; Quandt et al. 2004). It also documents pesticide exposure in nonagricultural communities (Barr et al. 2004; Berkowitz et al. 2003; Curl et al. 2003; Whyatt et al. 2003). Studies are beginning to examine the health effects of this pesticide exposure in communities (Alavanja et al. 2004; Eskenazi et al. 1999; Kamel and Hoppin 2004; Strong et al. 2004).

The exposure of migrant and seasonal farmworkers and their families to pesticides is an important research agenda. This agenda reflects concerns for ensuring social and environmental justice for this population (Arcury and Quandt 2003). Farmworkers experience high levels of occupational illness and injury (Villarejo 2003). They are exposed to high levels of pesticides at work, and they, their spouses, and their children are exposed to agricultural and residential pesticides at home (Arcury et al. 2005; Eskenazi et al. 2004; Fenske et al. 2000; McCauley et al. 2001; Quandt et al. 2004). Farmworkers have limited control over their work and residential environments (Austin et al. 2001), and they receive limited economic rewards for the high levels of environmental health risk inherent in their work (Carroll et al. 2005). Current regulations to protect farmworkers and their families from pesticide exposure are limited in scope, and these regulations are often ignored (Arcury et al. 2001a; U.S. General Accounting Office 2000).

Pesticide exposure research has been spurred on by the development of sensitive and reliable laboratory techniques that allow the detection of minute amounts of pesticides or pesticide metabolites in both environmental and biological media (e.g., Bravo et al. 2004; Geno et al. 1995, 1996; Olsson et al. 2004). However, these laboratory techniques are not widely available and are not always comparable across laboratories. Furthermore, published reports from investigators using the same analytic laboratories use different methods for displaying data, making comparisons among studies difficult (Wessels et al. 2003).

The power of research on farmworker pesticide exposure is limited because of variability in the collection of exposure data, the predictors of exposure considered, the laboratory procedures used in analyzing the exposure, and the measurement of exposure (Wessels et al. 2003). Within environmental exposure data, there is variability in the medium collected (e.g., dust vs. air) and in the way specific samples are collected (e.g., vacuum vs. wipes). Biomarker data are collected from urine and blood samples as well as meconium and cord blood. Variability in urine sample collection includes whether first morning void samples, spot samples, or total 24-hr voids are collected. Variability in the measurement of analytes is exemplified by the several ways in which studies assessing environmental exposure and biomarkers handle values that are below the limit of detection and samples with no detectable pesticide, how exposure variables are grouped for summary measures, and whether studies report results of urinary metabolites with or without adjustment for factors such as creatinine (Wessels et al. 2003).

The predictors of pesticide exposure (e.g., task, hygiene, proximity of housing to fields) used in analyses of farmworker pesticide exposure are dictated by the hypotheses being tested in a specific study. However, even when two studies attempt to measure the same predictor, they often do so in different ways. Having a basic set of predictors included in all studies would improve comparability. Finally, the specific health effects resulting from pesticide exposures are often ambiguous (Alavanja et al. 2004).

In summary, the sensitivity of laboratory techniques to measure pesticide exposure is increasing. Measuring pesticide exposure is important in populations, such as migrant and seasonal farmworkers, that are routinely exposed to pesticides but have limited power to control this exposure. However, studies of farmworker pesticide exposure have little comparability in data collection, data analysis, and data reporting related to environmental assessment, biomarkers, predictors of exposure and dose, and health outcomes from exposure. This lack of comparability limits the value of individual studies for understanding the proximal causes of exposure and for developing procedures to reduce this exposure. For these reasons, the Farmworker Pesticide Exposure Comparable Data Conference attempted to develop methodologic consensus in four areas of farmworker pesticide exposure research: environmental exposure assessment, biomarkers, personal and occupational predictors of exposure, and health outcomes of exposure.

## Challenges in Conducting Farmworker Pesticide Research

Research in farmworker populations must deal with a number of common challenges. These include defining the farmworker population, communicating with farmworkers, gaining access to the places farmworkers live and work, and the organization of agricultural work. Discussions during preparation for the conference, as well as at the conference reflected the effects of these common challenges on environmental pesticide exposure assessment, biomarkers, personal and occupational predictors of exposure, and health outcomes of exposure among farmworkers. A breakout session during the conference focused on these challenges. Because of the importance of these challenges for conducting farmworker pesticide research, each must be considered when reading the articles in this collection.

### Farmworkers in the United States

Knowing the number of farmworkers at risk is basic to toxicologic and epidemiologic analyses of pesticide exposure. However, there are several definitions for “farmworker,” and no source exists on the number of farmworkers.

Definitions of “farmworker” share specific key elements related to type of work, period of employment, and changing residence to engage in work. Using the definitions found in federal statutes governing migrant health funds, a migrant farmworker is an individual whose principal employment is in agriculture on a seasonal basis and who, for purposes of employment, establishes a temporary home. The migration may be from farm to farm, within a state, interstate, or international. A seasonal farmworker is an individual whose principal employment is in agriculture on a seasonal basis and who does not migrate.

Migrant and seasonal farmworkers work in at least 42 of the 50 states. National Agricultural Workers Survey data show that the national farmworker population became predominately Latino and Mexican in the 1990s (Mines et al. 1997). In 2002, 84% of migrant and seasonal farmworkers in the United States self-identified as Hispanic, and 75% of all farmworkers were born in Mexico (Carroll et al. 2005). Many of these farmworkers are legal residents of the United States; 25% are U.S. citizens, and 21% are legal permanent residents. Others are immigrants who come to the United States annually with H2A visas, which authorize nonimmigrant aliens to work in agricultural employment in the United States for a specified time period, normally less than 1 year. Finally, approximately half of farmworkers live in the United States without proper documentation. The lack of documentation often leads farmworkers to avoid contact with individuals whom they do not know, such as researchers, and keeps them from reporting pesticide exposure.

An accurate count of farmworkers in the United States is difficult to establish. In 1990, the Health Resources and Services Administration (1990) estimated that there were 4.2 million seasonal and migrant farmworkers and their dependents in the United States, with 1.6 million classified as migrant. In 2000, Larson (2000) prepared estimates of farmworkers in 10 states (Arkansas, California, Florida, Louisiana, Maryland, Mississippi, North Carolina, Oklahoma, Texas, and Washington) and reported the number of migrant farmworkers, seasonal farmworkers, and nonfarmworkers in farmworker households by county. Some state agencies provide estimates of the number of farmworkers who are employed in their state. Although different sources provide a general indication of the number of farmworkers in different geographical units, it is virtually impossible to establish the exact size of the farmworker population, particularly when dependents are considered.

### Mobility

Although many farmworkers are seasonal and do not change residence to work, many others are migrants. These migrant farmworkers will only be in particular locales during specific periods of each year. In 2001–2002, 42% of all farmworkers migrated. Of farmworkers who migrated, 38% were foreign-born newcomers, 30% international shuttle migrants, 5% international follow-the-crop migrants, 13% domestic shuttle migrants, and 14% domestic follow-the-crop migrants (Carroll et al. 2005).

In addition to the larger migration patterns, there is short-term mobility within local farmworker populations. Quandt et al. (2002) examined data on turnover in North Carolina residential sites to document the degree of mobility among farmworkers across a season and how this mobility can affect research. They found that approximately 30% of farmworkers changed residence over the course of the summer. Analysis of specific residence sites revealed both in- and out-migration. Although some sites were very stable, in others there was a complete turnover of residents one or more times across the agricultural season. Study designs must account for the residential fluidity of this population.

### Communication

Differences in language and limitations in education and literacy often complicate communication with individual farmworkers. Although some farmworkers are native English speakers and others speak a French Creole or a South Asian language, the great majority of farmworkers are Latino, and their primary language is Spanish (Carroll et al. 2005). Several linguistic characteristics make simple English-to-Spanish translation difficult. The Spanish spoken by farmworkers has national and regional dialects. The primary language of many “Latino” farmworkers is an indigenous (Native American) language, such as Mixteco or Tarasco (Alderete et al. 2000). For these farmworkers, Spanish is a second language, for which they may have limited facility (Arcury et al. 2001d; McCauley et al. 2001). Finally, the pesticide vocabulary of farmworkers includes both folk and Spanglish (“Spanishized” English) terms. For example, North Carolina farmworkers are more apt to refer to pesticides as “liquidos” than as “pesticidas,” and they often refer to applying pesticides at work with the term “sprayando” (English “spray,” Spanish “ando”), rather than “fumigando.”

Education level and literacy of the population also affect how researchers communicate with farmworkers. Farmworkers generally have fewer than 9 years of education (secondario level in the Mexican educational system) and often have fewer than 6 years (primario level in the Mexican system) (Arcury et al. 2001c; Kamel et al. 2001; McCauley et al. 2001). Literacy levels are also low; many farmworkers are functionally illiterate in Spanish and English.

Taken together, these characteristics make contacting, recruiting, and obtaining informed consent from farmworkers difficult. They also make the process of developing data collection forms and directions for collecting samples complex. Studies may include bias if 10% or more of the population must be excluded because they speak a language for which there are no qualified interpreters. All forms must be translated into Spanish, with the translation being educationally appropriate, including vernacular uses. Data collection may require personal interviews rather than self-administered questionnaires. When biological samples are collected, such as first morning void urine samples, directions for sample collection must be given verbally and reinforced with simple written directions.

### Access

Farmworkers constitute a hard-to-reach population that makes recruiting participants extremely difficult. There is no list of farmworkers from which to select a sample. Farmworkers often live in small groups that are scattered over large areas, often in camps that are located on unpaved roads miles from main thoroughfares. Many farmworkers do not want to be found and are hesitant to participate in any activity that appears to be official because they do not have documents. Often farm owners, growers, and crew leaders are reluctant to have farmworkers participate in research.

Access to farmworkers affects all aspects of research. How, where, and when farmworkers can be contacted and tracked for follow-up dictates how a research problem can be developed, as well as a project’s sample design and data collection. Access affects analysis by determining the hypotheses that can be tested and whether sufficient information can be collected for determining rates and predictors of exposure. Access also has ethical implications; investigators need to consider if they are placing farmworkers at risk for legal or workplace sanctions by recruiting them to a study.

Farmworkers can be accessed at a wide range of venues—farmworker residential camps, non-camp residences, clinics, unions, community organizations, community sites—each imposing its own limitations on research design. Access requires knowledge of these venues. For example, to access farmworkers at worksites, investigators must know which farms employ farmworkers and the periods during which farmworkers are employed. For access to residential camps, investigators must know their locations and when they are inhabited.

Each venue usually has a gatekeeper who controls access, and each gatekeeper may have reasons to restrict research. First and foremost, individual farmworkers control access to themselves and to their families at all venues. Even farmworkers with documents often may not want to be accessed because of fears of harassment from law enforcement agencies. Access to worksites and residential camps is controlled by growers, crew leaders, and unions. Access to worksites is often denied because neither grower nor crew leader wants work to stop. Growers may worry about liability that might result if researchers report that regulations are not being observed or that a worker is exposed to pesticides or gets sick from pesticide exposure.

Access to clinics and community organizations is controlled by their staffs. Clinicians are sometimes reluctant to participate in research because it can affect clinic flow and increase their costs. There is also often distrust for research based on the experiences of clinics and organizations with researchers who have not provided feedback or even acknowledged the help that they have received.

### Organization of work

The organization of work draws attention to the way that jobs are designed and performed (i.e., work processes) as well as the management, production methods, and human resource policies that shape work processes (National Institute for Occupational Safety and Health 2002). The organization of agricultural work, including its temporal structure, production methods, and dependence on contingent labor, create distinct challenges for conducting research on farmworker pesticide exposure.

Two aspects of the temporal structure of agricultural production, seasonality and the compressed time frame of production, introduce sampling, measurement, and data collection challenges. Different pesticides are used across the agricultural season. This heterogeneity introduces sampling and measurement challenges for the collection and laboratory analysis of environmental or biological samples and for documenting the tasks in which workers are involved. The narrow and compressed time frame within each phase of production further complicates pesticide exposure research because external forces (e.g., inclement weather) can have a dramatic effect on the research results.

Production methods, particularly degree of mechanization, introduce distinct challenges for pesticide exposure research. Mechanization influences how pesticide exposure might occur. When agricultural tasks rely on human labor, both inhalation and dermal routes of exposure may be common. However, inhalation may be the dominant route of exposure when production relies entirely on machinery. Mechanization can influence when exposure occurs, particularly when both machine and human labor are used in different phases of production. For example, potential exposure is delayed when machinery is used in planting and cultivating, but human labor is needed for harvesting.

Contingent employment relationships refer to “any job in which the worker does not have an explicit or implicit contract for long-term employment” (Polivka and Nardone 1989). All migrant and seasonal farmworkers are contingent workers. Agriculture’s heavy reliance on contingent employment relationships creates several challenges for designing and executing pesticide exposure research. First, the absence of long-term employment arrangements in farmwork undermines the ability to follow workers over time and limits researchers’ abilities to assess cumulative exposure to pesticides, the short- and long-term health consequences of pesticide exposure, and the effectiveness of interventions to prevent exposure. Use of day labor in some sectors of agriculture creates challenges for delineating whether pesticide exposure occurred as a result of farmwork or activities undertaken on another job (e.g., landscaping). Finally, the absence of long-term employment relationships creates difficulties in defining and creating representative samples of farmworkers because those in short-term relationships are difficult to identify and recruit into research studies, particularly if the day laborers are undocumented immigrants.

### Summary

Many characteristics of farmworkers—their number, mobility, language, education—present clear challenges for documenting pesticide exposure in this population. Characteristics of agricultural work further complicate pesticide exposure research designs. Investigators are discovering methods to overcome these challenges. Community-based participatory research designs in which communities actively collaborate with researchers are particularly helpful in this regard (Arcury et al. 2001b).

## Major Findings from Each of the Conference’s Working Groups

The conference working groups considered challenges in conducting farmworker pesticide research in developing their reports. The complete working group reports, as well as the cross-cutting report on laboratory and statistical analysis issues, are presented in the other articles of this mini-monograph. We summarize the major findings of these reports here.

### Environmental exposure assessment

Pesticide exposure assessment studies are conducted for a variety of purposes, and these purposes influence the sample collection and analysis strategies. All studies start with a basic framework of an exposure scenario that describes potential sources and pathways of exposures to individuals. Although the pesticides of interest and media sample vary based on the exposure scenario of interest and the agricultural practices in the region, efforts can be made to standardize how this information is presented so that researchers can compare across studies. To improve comparability across studies, investigators need to provide sufficient detail on the population sampled, the collection methodology, pesticides of interest, selection of sampling locations, chemical extraction, and analytical methods, as well as the statistical measures used to address values below the analytical detection limits. Presentation of this information will allow identification of commonalities and differences and aid in the identification of areas for comparable studies.

The most common environmental medium sampled to assess pesticide exposure among farmworkers and their families is dust. A variety of methods are employed to assess exposure to pesticides in dust, including hand wipes, surface wipes, and vacuum samples. Although surface loading is likely more closely related to exposure and health effects than is dust concentration, little research has been conducted on this topic, making results collected with different methods difficult to interpret. Thus, comparing the results of these two methods may be limited to rank ordering.

Understanding the exposure scenario is critical for evaluating farmworker exposure to pesticides. Although dust is believed to be one of the most important sources of exposure for farmworker families, little research has characterized the temporal and spatial variability of pesticides in the dust of farmworker homes. Additionally, few data are available from farmworkers regarding the assumptions used to construct exposure scenarios (e.g., hours spent in the home). Because farmworkers are often transient with regard to both location and occupation, gaining this information will be difficult. However, it is critical for the understanding of the long-term health effects of these exposures. Air, water, and food may also contribute to pesticide exposure among farmworkers; to date, much of the work has focused on pesticide residues and has relied on dust samples to reflect both carry-home exposure of the farmworker and potential exposures due to proximity to treated fields. Water is not anticipated to be an important exposure route for most pesticides, but exposure to pesticides from food has been poorly characterized, both in the general population and in farmworkers.

### Biomonitoring

Although biomonitoring has been used in occupational exposure assessment for more than 30 years, researchers are only beginning to understand the complexities involved with conducting appropriate biomonitoring exposure assessments and interpreting the resulting data. Biomonitoring has usually been applied to specific exposure scenarios with an anticipated potential exposure, for example, during application of pesticides. However, because many farmworkers work in fields in which data on the pesticide use and frequency are not readily available, the sample collection scheme can be complicated. Because most chemical exposures of concern for farmworker populations are short-lived, a single sampling during a nondefined exposure scenario may not adequately reflect the general exposure of the farmworker.

Blood and urine are the most frequently used matrices for exposure assessment using biomonitoring. The chemicals measured in these samples include the intact pesticides, their metabolites, and their environmental degradates. In many instances, a single measured analyte may represent exposure to more than one pesticide. Blood measurement usually offers more specificity; however, the levels are usually lower and much more difficult to detect. Relatively few methods exist to measure the multitude of pesticides that are currently being applied in the United States, and those that do exist are limited to only a few pesticides. For some pesticides or their metabolites, multiple laboratories make measurements, yet researchers have no firm grasp on how well the measurements from these laboratories agree. This specifically limits their ability to compare data across studies.

Few would argue that biological measurements add useful exposure assessment information to any study evaluating either predictors of exposure or resulting health outcomes. However, until there is a more uniform or standardized approach to using biomonitoring in exposure assessment, and until we have a better understanding of how to interpret the resulting data, its use may be limited.

### Predictors of exposure

Farmworkers are exposed to pesticides—or protected from exposure—by a range of factors inherent in their work and residential situations. These factors include behaviors such as the use of personal protective equipment as well as environmental characteristics such as the types of crops cultivated or the methods of pesticide application employed. A conceptual model was developed to identify the range of factors potentially associated with pesticide exposure among farmworkers and to propose a minimum set of measures necessary to understand farmworker pesticide exposure.

The model of farmworker pesticide exposure contrasts proximal and distal determinants of pesticide exposure. Proximal determinants of pesticide exposure include behaviors practiced either by farmworkers in the workplace or by farmworkers or their coresident household members at home, such as the use of personal protective equipment and laundry practices. These proximal factors are themselves determined by predictors considered more distal to the exposure. These include environmental conditions at work, at home, and in the larger community. These environmental factors affect exposure through behavior. The association of environmental and behavioral factors is moderated by psychosocial factors, including the attitudes, values, beliefs, and knowledge held by farmworkers.

Despite ongoing concern about pesticide exposure of farmworkers and their families, relatively few studies have tried to directly test the association of behavioral and environmental factors with pesticide exposure in this population. Most of the research to date has examined farmworker behaviors in the workplace. Recently, studies have begun to measure pesticides in farmworker residences, allowing the associations between household environmental factors and exposure to be evaluated. Although relationships between such characteristics of farmworkers as language and beliefs have been suggested, there have been no studies to evaluate these as predictors of pesticide exposure.

Limited evidence is available with which to understand how and why farmworkers and their families are actually exposed to pesticides. Future studies should attempt to use similar behavioral, environmental, and psychosocial measures to build a body of evidence with which to better understand the risk factors for pesticide exposure among farmworkers.

### Health outcomes of exposure

Numerous health conditions are associated with pesticide exposure. Organophosphates are associated with acute health problems such as nausea, dizziness, vomiting, headaches, abdominal pain, and skin and eye problems. Pesticide exposure is also associated with chronic health conditions or symptoms such as respiratory problems, memory disorders, dermatologic conditions, cancer, depression, neurologic deficits, miscarriages, and birth defects.

Little of the research on health effects associated with pesticide exposure has been conducted with farmworker populations. Several challenges for conducting epidemiologic studies with farmworkers have been discussed. Measurement of pesticide-related health outcomes faces additional challenges. Many states do not have pesticide poisoning reporting systems. Clinicians often lack the knowledge to recognize the symptoms associated with acute pesticide exposure. Farmworkers are unlikely to be represented in workers compensation and health insurance databases or disease registries because of the nature of agricultural work and their access to care. These are significant issues that severely affect the ability to measure the health of this population.

Neurobehavioral performance batteries are a well-recognized method of assessing potential health effects associated with pesticide exposure. Studies examining neurobehavioral performance have found that pesticide exposure is associated with deficits in cognitive and psychomotor function (Kamel and Hoppin 2004). However, use of these batteries with non-English-speaking immigrant populations such as farmworkers presents additional challenges. Significant information is available to optimize the capacity of researchers to use these batteries and comparisons can be made across studies. Discrepancies that are seen may be attributed to exposure differences across agricultural work settings and how exposure is defined.

The incorporation of biomarkers of health effects in study designs offers new insight into the mechanisms of pesticide toxicity and early indicators of biological effects. Acetyl-cholinesterase is extensively used as a biomarker for exposure to organophosphate pesticides in farmworker populations. It is especially useful among workers expected to be at the greatest risk for acute exposure. New techniques are being used with farmworkers, including biomarkers of DNA damage and markers of cellular reaction. Reviews of studies to date point to the capacity of acute pesticide exposure and chronic low-dose exposure to affect the rates of DNA damage. A limitation of these markers is that the indication of a marker cannot be translated into a specific risk of disease. Gene expression studies are an emerging area that has the potential to greatly increase understanding of the health risks associated with pesticide exposure.

The capacity to document the extent of pesticide exposure is far greater than the ability to measure health effects. There is a critical need to link studies of exposure to pesticides to investigations of potential health effects, and investigators should be encouraged to incorporate biological markers in their study designs. Many studies of farmworkers suffer from small sample sizes. Cross-study comparisons can be made if standardized measures such as neurobehavioral batteries are used. Meta-analyses may help in quantifying the health risks associated with pesticide exposure in this population as more standardized measures are used.

### Laboratory and statistical analysis issues

Statistical issues are fundamental to study design and interpretation. A tremendous number of different statistical issues arise in the analysis of farmworker pesticide exposures and related data. These issues can be generally categorized as relating to *a*) sampling and design; *b*) missing or misclassified data and subsequent biases; *c*) data reporting and analytical reliability issues, which include laboratory data quality; *d*) extensions to standard statistical models and analyses (e.g., primarily inferential statistical issues); and *e*) post-hoc analyses and combining study results in which cross-laboratory comparisons may be required. There appears to be no standard approach for addressing these issues in farmworker studies. Statistical or laboratory issues should be considered in each component of research design. For example, the number of samples that should be collected and the time intervals at which they should be collected must be considered. With very little knowledge of intra- and interperson variability in sample measurements, the sampling strategy is not straightforward. The treatment of values below the limit of detection is not standard, with many different approaches used in the literature. The study outcomes may depend somewhat upon how these data are treated. Assessment of confidence in laboratory measurements and defining issues of measurement quality must be considered. By adopting a more standard approach to deal with these issues, researchers may be better able to integrate data among studies.

## Conclusions

The articles in this mini-monograph document the challenges to understanding the environmental and occupational health risk of pesticide exposure for migrant and seasonal farmworkers. There has been considerable progress in measuring the environmental and biological exposure of farmworkers to pesticides, of documenting the relationship of specific environmental and behavioral predictors of farmworker pesticide exposure, and of linking farmworker pesticide exposure to health outcomes. However, although current laboratory methods are capable of providing detailed measures of environmental pesticide exposure and biological exposure, several methodologic issues need to be resolved to improve the ability to document the types and amounts of pesticides to which farmworkers are exposed. Each of the articles in this mini-monograph makes recommendations to improve farmworker pesticide exposure research. These pose challenges that researchers must address to develop ways to better protect the health of farmworkers.

## References

[b1-ehp0114-000923] Acquavella JF, Alexander BH, Mandel JS, Gustin C, Baker B, Chapman P (2004). Glyphosate biomonitoring for farmers and their families: results from the Farm Family Exposure Study. Environ Health Perspect.

[b2-ehp0114-000923] Alavanja MCF, Hoppin JA, Kamel F (2004). Health effects of chronic pesticide exposure: cancer and neurotoxicity. Annu Rev Public Health.

[b3-ehp0114-000923] Alderete E, Vega WA, Kolody B, Aguilar-Gaxiola S (2000). Lifetime prevalence of and risk factors for psychiatric disorders among Mexican migrant farmworkers in California. Am J Public Health.

[b4-ehp0114-000923] Arcury TA, Quandt SA (2003). Pesticides at work and at home: exposure of migrant farmworkers and their families. Lancet.

[b5-ehp0114-000923] Arcury TA, Quandt SA, Cravey AJ, Elmore RC, Russell GB (2001a). Farmworker reports of pesticide safety and sanitation in the work environment. Am J Ind Med.

[b6-ehp0114-000923] Arcury TA, Quandt SA, Dearry A (2001b). Farmworker pesticide exposure and community-based participatory research: rationale and practical applications. Environ Health Perspect.

[b7-ehp0114-000923] Arcury TA, Quandt SA, Preisser JS (2001c). Predictors of illness incidence and prevalence of green tobacco sickness among Latino farmworkers in North Carolina, U.S.A. J Epidemiol Community Health.

[b8-ehp0114-000923] Arcury TA, Quandt SA, Preisser JS, Norton D (2001d). The incidence of green tobacco sickness among Latino farmworkers. J Occup Environ Med.

[b9-ehp0114-000923] Arcury TA, Quandt SA, Rao P, Doran AM, Snively BM, Barr DB (2005). Organophosphate pesticide exposure in farmworker family members in western North Carolina and Virginia: case comparisons. Hum Organ.

[b10-ehp0114-000923] Austin CK, Arcury TA, Quandt SA, Preisser JS, Saavedra RM, Cabrera LF (2001). Training farmworkers about pesticide safety: issues of control. J Health Care Poor Underserved.

[b11-ehp0114-000923] Barr DB, Bravo R, Weerasekera G, Caltabiano LM, Whitehead RD, Olsson AO (2004). Concentrations of dialkylphosphate metabolites of organophosphorus pesticides in the U.S. population. Environ Health Perspect.

[b12-ehp0114-000923] Berkowitz GS, Obel J, Deych E, Lapinski R, Godbold J, Liu Z (2003). Exposure to indoor pesticides during pregnancy in a multiethnic, urban cohort. Environ Health Perspect.

[b13-ehp0114-000923] Bravo R, Caltabiano LM, Weerasekera G, Whitehead RD, Fernandez C, Needham LL (2004). Measurement of dialkyl phosphate metabolites of organophosphorus pesticides in human urine using lyophilization with gas chromatography-tandem mass spectrometry and isotope dilution quantification. J Expo Anal Environ Epidemiol.

[b14-ehp0114-000923] Carroll DJ Samardick R Bernard S Gabbard S Hernandez T 2005. Findings from the National Agricultural Workers Survey (NAWS) 2001–2002: A Demographic and Employment Profile of United States Farm Workers. Research Report No. 9. Washington, DC:U.S. Department of Labor, Office of the Assistant Secretary for Policy, Office of Programmatic Policy.

[b15-ehp0114-000923] Curl CL, Fenske RA, Elgethun K (2003). Organophosphorus pesticide exposure of urban and suburban preschool children with organic and conventional diets. Environ Health Perspect.

[b16-ehp0114-000923] Curl CL, Fenske RA, Kissel JC, Shirai JH, Moate TF, Griffith W (2002). Evaluation of take-home organophosphorus pesticide exposure among agricultural workers and their children. Environ Health Perspect.

[b17-ehp0114-000923] Eskenazi B, Bradman A, Castorina R (1999). Exposures of children to organophosphate pesticides and their potential adverse health effects. Environ Health Perspect.

[b18-ehp0114-000923] Eskenazi B, Harley K, Bradman A, Weltzien E, Jewell NP, Barr DB (2004). Association of *in utero* organophosphate pesticide exposure and fetal growth and length of gestation in an agricultural population. Environ Health Perspect.

[b19-ehp0114-000923] Fenske RA, Lu C, Barr D, Needham L (2002). Children’s exposure to chlorpyrifos and parathion in an agricultural community in central Washington State. Environ Health Perspect.

[b20-ehp0114-000923] Fenske RA, Lu C, Simcox NJ, Loewenherz C, Touchstone J, Moate TF (2000). Strategies for assessing children’s organophosphorus pesticide exposures in agricultural communities. J Expo Anal Environ Epidemiol.

[b21-ehp0114-000923] Geno PW, Camann DE, Harding HJ, Villalobos K, Lewis RG (1996). Handwipe sampling and analysis procedure for the measurement of dermal contact with pesticides. Arch Environ Contam Toxicol.

[b22-ehp0114-000923] Geno PW Majumda TK Camann DE Bond AE 1995. A multiresidue GC/MS method for the determination of pesticides in environmental media. In: Measurement of Toxic and Related Air Pollutants. Publication VIP-50. Pittsburgh, PA:Air and Waste Management Association, 531–541.

[b23-ehp0114-000923] Health Resources and Services Administration. 1990. An Atlas of State Profiles Which Estimates Number of Migrant and Seasonal Workers and Members of their Families. Washington, DC:U.S. Department of Health and Human Services, Public Health Service, Migrant Health Branch.

[b24-ehp0114-000923] Kamel F, Hoppin JA (2004). Association of pesticide exposure with neurologic dysfunction and disease. Environ Health Perspect.

[b25-ehp0114-000923] Kamel F, Moreno T, Rowland AS, Stallone L, Ramirez-Garnica G, Sandler DP (2001). Recruiting a community sample in collaboration with farmworkers. Environ Health Perspect.

[b26-ehp0114-000923] Lambert WE, Lasarev M, Muniz J, Scherer J, Rothlein J, Santana J (2005). Variation in organophosphate pesticide metabolites in urine of children living in agricultural communities. Environ Health Perspect.

[b27-ehp0114-000923] Larson AC 2000. Migrant and Seasonal Farmworker Enumeration Profiles Study. Final. Prepared for the Migrant Health Program, Bureau of Primary Health Care, Health Resources and Services Administration. Vashon Island, WA:Larson Assistance Services. Available: http://bphc.hrsa.gov/migrant/Enumeration/EnumerationStudy.htm/ [accessed 15 November 2004].

[b28-ehp0114-000923] Lu C, Fenske RA, Simcox NJ, Kalman D (2000). Pesticide exposure of children in an agricultural community: evidence of household proximity to farmland and take home exposure pathways. Environ Res.

[b29-ehp0114-000923] Mandel JS, Alexander BH, Baker B, Acquavella JF, Chapman P, Honeycutt R (2005). Biomonitoring for farm families: farm family exposure study. Scand J Work Environ Health.

[b30-ehp0114-000923] McCauley LA, Lasarev MR, Higgins G, Rothlein J, Muniz J, Ebbert C (2001). Work characteristics and pesticide exposures among migrant agricultural families: a community-based research approach. Environ Health Perspect.

[b31-ehp0114-000923] Mines R Gabbard S Steirman A 1997. A Profile of U.S. Farm Workers: Demographics, Household Composition, Income and Use of Services. Research Report No. 6. Washington, DC:U.S. Department of Labor.

[b32-ehp0114-000923] National Institute for Occupational Safety and Health. 2002. The Changing Organization of Work and the Safety and Health of Working People: Knowledge Gaps and Research Directions. DHHS Publ no 2002–116. Cincinnati, OH:National Institute for Occupational Safety and Health.

[b33-ehp0114-000923] Olsson AO, Baker SE, Nguyen JV, Romanoff LC, Udunka SO, Walker RD (2004). A liquid chromatography-tandem mass spectrometry multiresidue method for quantification of specific metabolites of organophosphorus pesticides, synthetic pyrethroids, selected herbicides, and DEET in human urine. Anal Chem.

[b34-ehp0114-000923] Polivka AE, Nardone T (1989). On the definition of “contingent work. Mon Labor Rev.

[b35-ehp0114-000923] Quandt SA, Arcury TA, Rao P, Snively BG, Camann DE, Doran AM (2004). Agricultural and residential pesticides in wipe samples from farmworker family residences in North Carolina and Virginia. Environ Health Perspect.

[b36-ehp0114-000923] Quandt SA, Preisser JS, Arcury TA (2002). Mobility patterns of migrant farmworkers in North Carolina: implications for occupational health research and policy. Hum Organ.

[b37-ehp0114-000923] Strong LL, Thompson B, Coronado GD, Griffith WC, Vigoren EM, Islas I (2004). Health symptoms and exposure to organophosphate pesticides in farmworkers. Am J Ind Med.

[b38-ehp0114-000923] U.S. General Accounting Office. 2000. Pesticides: Improvements Needed to Ensure the Safety of Farmworkers and Their Children. GAO/RCED-00–40. Washington, DC:U.S. General Accounting Office.

[b39-ehp0114-000923] Villarejo D (2003). The health of U.S. hired farm workers. Annu Rev Public Health.

[b40-ehp0114-000923] Wessels D, Barr DB, Mendola P (2003). Use of biomarkers to indicate exposure of children to organophosphate pesticides: implications for a longitudinal study of children’s environmental health. Environ Health Perspect.

[b41-ehp0114-000923] Whyatt RM, Barr DB, Camann DE, Kinney PL, Barr JR, Andrews HF (2003). Contemporary-use pesticides in personal air samples during pregnancy and blood samples at delivery among urban minority mothers and newborns. Environ Health Perspect.

